# Supplementary material for: Taphonomic and Diagenetic Pathways to Protein Preservation, Part I: The Case of Tyrannosaurus rex Specimen MOR 1125
Source: Biology (Basel). 2021 Nov 17;10(11):1193. doi: 10.3390/biology10111193 (PMC8614911; doi:10.3390/biology10111193)
Supplement: Supplementary file 1 [file biology-10-01193-s001.zip › Ullmann_et_al_Supp_File_1_FINAL-BrexREVISED.pdf]

SUPPORTING INFORMATION

Biology

Taphonomic and diagenetic pathways to protein preservation, part I: the case of *Tyrannosaurus rex* specimen MOR 1125

Paul V. Ullmann[1,*], Kyle Macauley[1], Richard D. Ash[2], Ben Shoup[3], John B. Scannella[4,5]

[1]Department of Geology, Rowan University, Glassboro, NJ 08028, USA

[2]Department of Geology, University of Maryland, College Park, MD 20742, USA

[3]Absaroka Energy & Environmental Solutions, Buffalo, WY 82834, USA

[4]Museum of the Rockies, Montana State University, Bozeman, MT 59717, USA

[5]Department of Earth Sciences, Montana State University, Bozeman, MT 59717, USA

*Correspondence: ullmann@rowan.edu

This supplement provides: (1) further details on the sedimentology of the entombing sandstone and overlying Hell Creek Formation strata within the MOR 1125 quarry; (2) additional details on our LA-ICPMS methods; (3) discussion of potential tetrad effects in the right femur of MOR 1125; (4) further discussion of intra-bone fractionation trends in $(La/Yb)_N$ versus $(La/Sm)_N$ plots; (5) discussion of potential sequestration process that may have limited REE availability to the bones of MOR 1125; (6) sources for environmental data in Figure 7 of the main text; (7) the full stratigraphic section across the entire butte from which MOR 1125 was collected (Figure S1), and; (8) additional data on the trace element composition of the femur of MOR 1125 (Figures S2, S3). Raw transect data are provided separately in Data S1 as an Excel XLSX file.

Further Details on the Sedimentology and Stratigraphy of the MOR 1125 Quarry

Beds within the sandstone entombing MOR 1125 are comprised of thin sets containing thick (~3–5 cm) cosets that dip from 6–24° in various directions, but mainly to the southeast. A thin (~8 cm) interbed of laminated siltstone, rich in large and taxonomically-diverse fossil leaves, occurs within these coarse horizons and pinches out to the north within the quarry. Silty rip-up clasts and thin, organic-rich inclined heterolithic strata occur occasionally throughout the sands immediately overlying the fossil bones, and a large, partially-coalified log was found ~30 cm over a number of the bones in the center of the quarry. A femur and a few fragments of turtle carapace of an indeterminate taxon were also found within the entombing sandstone, as well as an ilium and a few partial vertebrae referable to *Thescelosaurus* and the astragalus of a smaller theropod dinosaur.

Strata exposed in the upper portion of the quarry wall begin with a thin (~15 cm) sequence of organic-rich fine sand to muddy inclined heterolithic strata containing occasional burrows and root traces, followed up-section by 1.1 m of trough cross-bedded medium sands that contain rare, discontinuous mud stringers (Figure 3A). In the southeast portion of the quarry, sets within these sandstones are ~2 cm thick and cosets dip ~16° to the southwest. Above these deposits lie: (1) a thick sequence (~12 m) of interbedded, gray, organic-rich shales and massive mudstones, which include a greenish-gray horizon with abundant root traces, followed by; (2) 11.7 m of fine to medium-grained, litharenitic, trough cross-bedded and ripple-stratified sandstone containing abundant and closely-spaced, organic-rich inclined heterolithic strata near its top (Figure S1). These represent, respectively, floodplain mudstones and channel sandstones that are typical of the middle (M3) and upper (U3) units of the Hell Creek Formation [1–3]. The highest stratum exposed in the butte consists of ~10 m of massive, calcareous, litharenitic, fine-grained sandstone (Figure S1).

LA-ICPMS Methodology

LA-ICPMS analyses were conducted using a New Wave UP-213 (213 nm wavelength) Nd:YAG laser coupled to a Finnigan Element2 ICPMS at the University of Maryland. The laser was operated at 2–3 J/cm^2 and a pulse rate of 7 Hz. Transect data were collected using a laser diameter of 30 μm moving at a scan speed of 50 μm/s, and background collection was performed prior to each reading for 20 s. NIST 610 glass was used as an external standard and elemental concentrations were calculated based on normalization to 55.8% CaO in bone apatite. NASC-normalized REE ratios were used to calculate $(Ce/Ce^*)_N$, $(Ce/Ce^{**})_N$, $(Pr/Pr^*)_N$, and $(La/La^*)_N$

anomalies following Herwartz et al. [4]: $(Ce/Ce^*)_N = Ce_N/(0.5*La_N + 0.5*Pr_N)$, $(Ce/Ce^{**})_N = Ce_N/(2*Pr_N – Nd_N)$, $(Pr/Pr^*)_N = Pr_N/(0.5*Ce_N + 0.5*Nd_N)$, and $(La/La^*)_N = La_N/(3*Pr_N – 2*Nd_N)$.

Potential Tetrad Effects in MOR 1125

Lack of MREE enrichment causes transects across the cortex of the femur of MOR 1125 to lack distinct W-shape profiles indicative of tetrad effects (unique elemental behavior based on ionic charge, radius, and configuration of the outer electron shell; [4,5]) or extensive secondary apatite precipitation [4]. However, a few transects across the middle and internal cortices display potential evidence of weak tetrad effects in the form of subtle W-shape NASC normalized patterns (Figure 6B) and/or positive Y/Ho anomalies (Figure S2; cf. [4]). According to Herwartz et al. [4], these trends may reflect either: (1) tetrad effects during uptake; (2) fractionation during uptake, in which LREE are preferentially adsorbed by the external-most cortex (resulting in changing of the pore fluid composition as it percolates deeper into the bone), and/or; (3) incorporation of elemental readings from secondarily precipitated (authigenic) phosphate which has scavenged trace elements from the pore fluid (i.e., during recrystallization of bone crystallites into larger, more diagenetically-stable fluorapatite crystals). Although it is difficult to tease apart the relative importance of these potential causes, the complete lack of any signs of double medium diffusion (*sensu* [6]) through Haversian canals in addition to advective diffusion in concentration-depth profiles (Table 2) implies that fractionation during uptake was likely a major contributing factor. Abundant other data (normal intra-bone fractional trends in the spider diagram [Figure 6B], the flatter profile for U than REE despite their similar diffusivities [Figure

4A,B], low concentrations of MREE and elements with low-moderate diffusivities in middle cortices [Figure 4A,B, Data S1]) also signify fractionation during uptake and changes in the pore fluid composition over time. Therefore, the positive Y/Ho anomalies observed in the middle and internal cortices of MOR 1125 may reflect this typical "fossilization" process more so than tetrad effects.

Intra-Bone Fractionation in $(La/Yb)_N$ vs. $(La/Sm)_N$

The pattern seen in the femur of MOR 1125 of decreasing $(La/Yb)_N$ yet unchanging $(La/Sm)_N$ with cortical depth (Figure 7B of main text) has been interpreted many ways in the literature. For example, Reynard et al. [7] assigned such patterns to represent "adsorption-dominated" REE incorporation mechanics, whereas Trueman et al. [8] interpreted such specimens (their 'group 3') to reflect uptake of REE from fluvially-derived pore fluids. Most recently, Herwartz et al. [4] reconsidered $(La/Yb)_N$–$(La/Sm)_N$ patterns in light of new laser-ablation derived data and concluded that all previous interpretations based on solution ICPMS analyses (e.g., [7,8]) were biased by bulk sample averaging effects. Herwartz et al. [4] (p. 176) (and Trueman et al. [9]) offer a compelling argument that such ratio trends reflect intra-bone fractionation driven by decreasing partition coefficients with increasing atomic radii for REE; because the order of closest fit of REE ionic radii to the Ca^{2+} lattice site in bone hydroxyapatite is $Sm^{3+} > La^{3+} > Yb^{3+}$, fractionation based on ionic radius will occur during uptake, causing preferential earlier uptake of Sm and La by the external cortex because of their better fit. This "normal" fractionation would thus depress $(La/Yb)_N$ values in the internal cortex relative to the external cortex, as seen in MOR 1125 (Figure 7B).

Processes Which Potentially Limited REE Availability

Complexation of dissolved REE with carbonate anions is common in natural waters [10,11], especially in coastal fresh and brackish waters [12,13] due to chemical weathering [14] and the decomposition of abundant organic matter in coastal sediments [15]. Given the sedimentologic, stratigraphic, and geochemical indications that MOR 1125 was buried in an estuarine channel (see below for geochemical support), it is therefore plausible that extensive complexation may have partially suppressed the availability of trace elements during early diagenesis. Humic acids released during the decay of plant debris in the surrounding sediments and of any residual dinosaur tissues (e.g., muscle) may also have formed complexes with dissolved REE [16], further limiting their availability for uptake by the bone. Dissolved phosphates released from decaying organic matter [17–19] may also have scavenged REE ions from solution via coprecipitation within trace secondary phosphates in the surrounding sediments (cf. [20,21]). We were not able to test quarry sediments for trace phosphatic phases in this study, but they are almost certainly present as lowland/coastal watersheds generally contain more phosphate than upstream regions [18,22]. In the absence of such direct tests, these potential sequestration processes (carbonate complexation and/or coprecipitation with phosphate) remain speculative, but the depositional setting implies that each may have contributed to limiting the supply of trace elements within surrounding early-diagenetic pore fluids.

Sources for Environmental Data in Figure 7

Literature data for environmental samples in Figure 7A of the main text are as follows: river waters (green field; [23–42]); suspended river loads (dull pink field; [24,43]); groundwaters (bright pink field; [29,44–52]); lakes (purple field; [29,32,33,36,45,48,53,54]); estuaries (yellow field; [25,51,55–57]); coastal waters (light blue field; [23,25,26,37,58,59]); seawater (dark blue field; [60–78]); sea floor particles (gray field; [64,72]); marine pore fluids (orange field; [58,76,79,80]).

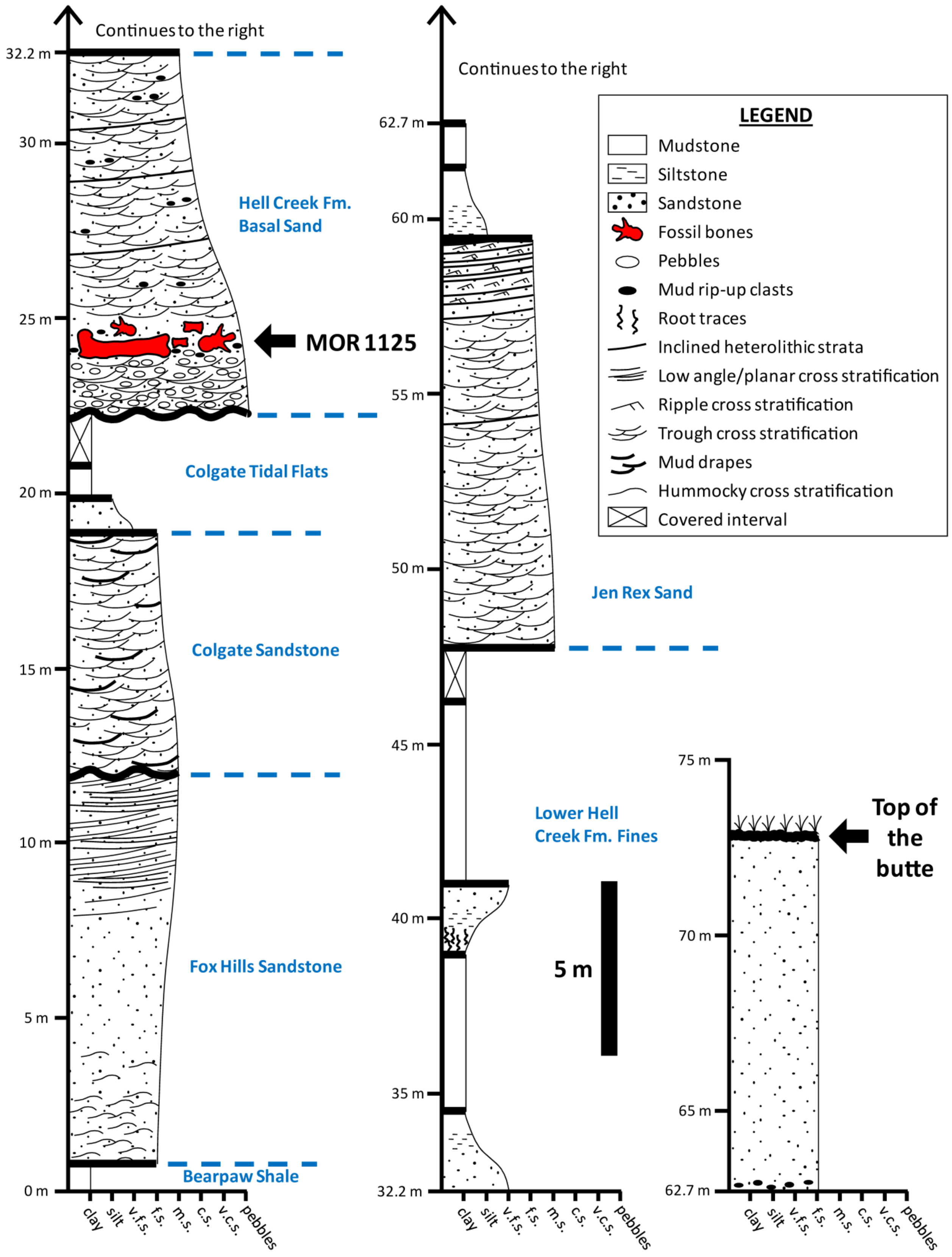

Continues to the right
32.2 m
30 m
25 m
20 m
15 m
10 m
5 m
0 m
Hell Creek Fm.
Basal Sand
MOR 1125
Colgate Tidal Flats
Colgate Sandstone
Fox Hills Sandstone
Bearpaw Shale
clay
silt
v.f.s.
f.s.
m.s.
c.s.
v.c.s.
pebbles
Continues to the right
62.7 m
60 m
55 m
50 m
45 m
40 m
35 m
32.2 m
Jen Rex Sand
Lower Hell
Creek Fm. Fines
5 m
LEGEND
Mudstone
Siltstone
Sandstone
Fossil bones
Pebbles
Mud rip-up clasts
Root traces
Inclined heterolithic strata
Low angle/planar cross stratification
Ripple cross stratification
Trough cross stratification
Mud drapes
Hummocky cross stratification
Covered interval
75 m
70 m
65 m
62.7 m
Top of
the
butte

Figure S1. Complete stratigraphic section across the entire butte from which MOR 1125 was collected. Abbreviations: c.s., coarse sand; f.s., fine sand; m.s., medium sand; v.c.s., very coarse sand; v.f.s., very fine sand. Scale bar as indicated. Lithostratigraphic assignments and unit names (in blue text) follow the revisions of those from Hartman et al. [2] by Fowler [3].

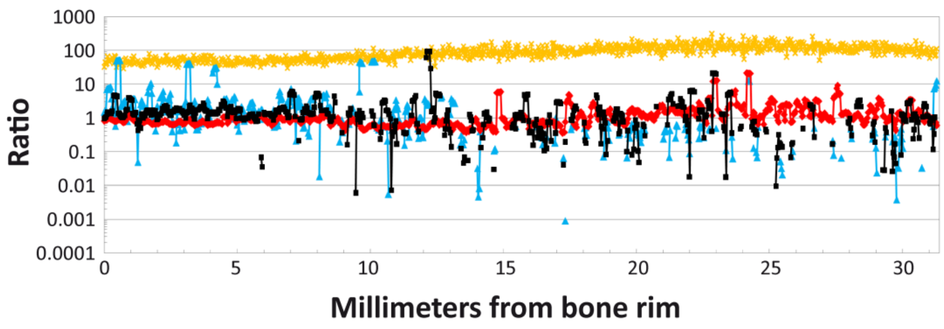

Figure S2. Intra-bone patterns of $(Ce/Ce^*)_N$, $(Ce/Ce^{**})_N$, and $(La/La^*)_N$ anomalies and Y/Ho ratios within the femur of MOR 1125. $(Ce/Ce^*)_N$ values (red curves), $(Ce/Ce^{**})_N$ values (black curves), and $(La/La^*)_N$ anomalies (blue curves) were calculated as outlined in the Materials and Methods. Y/Ho ratio data are presented as the orange curve. Absence of $(Ce/Ce^*)_N$, $(Ce/Ce^{**})_N$, and $(La/La^*)_N$ anomalies occurs at 1.0.

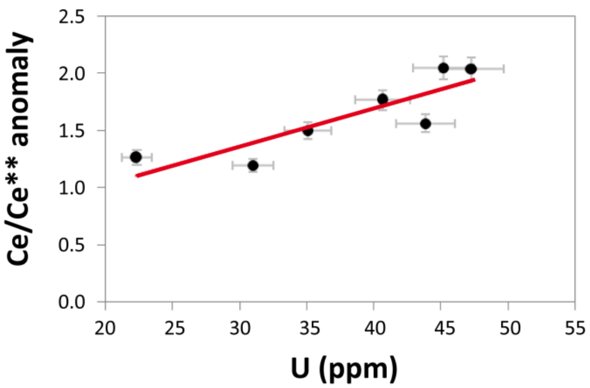

Figure S3. Cerium anomaly $(Ce/Ce^{**})_N$ values plotted against uranium (U) concentrations in the femur of MOR 1125. Absence of the cerium anomaly is at a value of 1.0. Error bars, in gray, are based on analytical reproducibility of ± 5%. Note the positive correlation ($r^2 = 0.75$), suggesting similar timescales for uptake of U and Ce (according to [81]).

REFERENCES

1) Horner, J.R.; Goodwin, M.B.; Myhrvold, N. Dinosaur census reveals abundant *Tyrannosaurus* and rare ontogenetic stages in the Upper Cretaceous Hell Creek Formation (Maastrichtian), Montana, USA. *PLoS ONE* **2011**, *6*, e16574.

2) Hartman, J.H.; Butler, R.D.; Weiler, M.W.; Schumaker, K.K. Context, naming and formal designation of the Cretaceous Hell Creek Formation lectostratotype, Garfield County, Montana. In *Through the end of the Cretaceous in the type locality of the Hell Creek Formation in Montana and adjacent areas*; Wilson, G.P.; Clemens, W.A.; Horner, J.R.; Hartman, J.H., Eds.; Geological Society of America Special Paper 503: Boulder, CO, USA, **2014**; pp. 89–121

3) Fowler, D. The Hell Creek Formation, Montana: a stratigraphic review and revision based on a sequence stratigraphic approach. *Geosciences* **2020**, *10*, 435.

4) Herwartz, D.; Tütken, T.; Jochum, K.P.; Sander, P.M. Rare earth element systematics of fossil bone revealed by LA-ICPMS analysis. *Geochim. Cosmochim. Ac*. **2013**, *103*, 161–183.

5) Hinz, E.A.; Kohn, M.J. The effect of tissue structure and soil chemistry on trace element uptake in fossils. *Geochim. Cosmochim. Ac*. **2010**, *74*, 3213–3231.

6) Kohn, M.J. Models of diffusion-limited uptake of trace elements in fossils and rates of fossilization. *Geochim. Cosmochim. Ac*. **2008**, *72*, 3758–3770.

7) Reynard, B.; Lecuyer, C.; Grandjean, P. Crystal-chemical controls on rare-earth element concentrations in fossil biogenic apatites and implications for paleoenvironmental reconstructions. *Chem. Geol*. **1999**, *155*, 233–241.

8) Trueman, C.N.; Behrensmeyer, A.K.; Potts, R.; Tuross, N. High-resolution records of location and stratigraphic provenance from the rare earth element composition of fossil bones. *Geochim. Cosmochim. Ac*. **2006**, *70*, 4343–4355.

9) Trueman, C.N.; Kocsis, L.; Palmer, M.R.; Dewdney, C. Fractionation of rare earth elements within bone mineral: a natural cation exchange system. *Palaeogeogr. Palaeocl*. **2011**, *310*, 124–132.

10) Cantrell, K.J.; Byrne, R.H. Rare earth element complexation by carbonate and oxalate ions. *Geochim. Cosmochim. Ac*. **1987**, *51*, 597–605.

11) Luo, Y.-R.; Byrne, R.H. Carbonate complexation of yttrium and the rare earth elements in natural waters. *Geochim. Cosmochim. Ac*. **2004**, *68*, 691–699.

12) Livingstone, D.A. Chemical composition of rivers and lakes. In *Data of Geochemistry*; Fleischer, M., Ed.; USGS: Washington, DC, USA, **1963**; pp. G1–G64.

13) Zhang, J.; Létolle, R.; Jusserand, C. Major element chemistry of the Huanghe (Yellow River), China – weathering processes and chemical fluxes. *J. Hydrol.* **1995**, *168*, 173–203.

14) Stallard, R.F.; Edmond, J.M. Geochemistry of the Amazon 3. Weathering chemistry and limits to dissolved inputs. *J. Geophys. Res.* **1987**, *92*, 8293–8302.

15) Berner, R.A. Calcium carbonate concretions formed by the decomposition of organic matter. *Science* **1968**, *159*, 195–197.

16) Pourret, O.; Davranche, M.; Gruau, G.; Dia, A. Rare earth elements complexation with humic acid. *Chem. Geol*. **2007**, *243*, 128–141.

17) Allison, P.A. Konservat-Lagerstätten: cause and classification. *Paleobiology* **1988**, *14*, 331–344.

18) Föllmi, K.B. The phosphorous cycle, phosphogenesis and marine phosphate-rich deposits. *Earth-Sci. Rev.* **1996**, *40*, 55–124.

19) Wang, L.; Liu, Q.; Hu, C.; Liang, R.; Qiu, J.; Wang, Y. Phosphorous release during decomposition of the submerged macrophyte *Potamogeton crispus*. *Limnology* **2018**, *19*, 355–366.

20) Byrne, R.H.; Liu, X.; Schijf, J. The influence of phosphate coprecipitation on rare earth distributions in natural waters. *Geochim. Cosmochim. Ac*. **1996**, *60*, 3341–3346.

21) Liu, X.; Byrne, R.H.; Schijf, J. Comparative coprecipitation of phosphate and arsenate with yttrium and the rare earths: the influence of solution complexation. *J. Solution Chem.* **1997**, *26*, 1187–1198.

22) Brunet, R.-C.; Astin, K.B. Variation in phosphorus flux during a hydrological season: the River Ardour. *Wat. Res.* **1998**, *32*, 547–558.

23) Hoyle, J.; Elderfield, H.; Gledhill, A.; Greaves, M. The behaviour of the rare earth elements during mixing of river and sea waters. *Geochim. Cosmochim. Ac*. **1984**, *48*, 143–149.

24) Goldstein, S.J.; Jacobsen, S.B. Rare earth elements in river waters. *Earth Planet. Sc. Lett.* **1988**, *89*, 35–47.

25) Elderfield, H.; Upstill-Goddard, R.; Sholkovitz, E.R. The rare earth elements in rivers, estuaries, and coastal seas and their significance to the composition of ocean waters. *Geochim. Cosmochim. Ac*. **1990**, *54*, 971–991.

26) Bau, M.; Dulski P. Anthropogenic origin of positive gadolinium anomalies in river waters. *Earth Planet. Sc. Lett*. **1996**, *143*, 245–255.

27) Biddau, R.; Cidu, R.; Frau, F. Rare earth elements in waters from the albitite-bearing granodiorites of Central Sardinia, Italy. *Chem. Geol*. **2002**, *182*, 1–14.

28) Åström, M.; Corin, N. Distribution of rare earth elements in anionic, cationic and particulate fractions in boreal humus-rich streams affected by acid sulphate soils. *Water Res.* **2003**, *37*, 273–280.

29) Bwire Ojiambo, S.; Lyons, W.B.; Welch, K.A.; Poreda, R.J.; Johannesson, K.H. Strontium isotopes and rare earth elements as tracers of groundwater-lake water interactions, Lake Naivasha, Kenya. *Appl. Geochem.* **2003**, *18*, 1789–1805.

30) Tang, J.; Johannesson, K.H. Speciation of rare earth elements in natural terrestrial waters: assessing the role of dissolved organic matter from the modeling approach. *Geochim. Cosmochim. Ac*. **2003**, *67*, 2321–2339.

31) Centeno, L.M.; Faure, G.; Lee, G.; Talnagi, J. Fractionation of chemical elements including the REEs and ^{226}Ra in stream contaminated with coal-mine effluent. *Appl. Geochem.* **2004**, *19*, 1085–1095.

32) Johannesson, K.H.; Tang, J.; Daniels, J.M.; Bounds, W.J.; Burdige, D.J. Rare earth element concentrations and speciation in organic-rich blackwaters of the Great Dismal Swamp, Virginia, USA. *Chem. Geol*. **2004**, *209*, 271–294.

33) Gammons, C.H.; Wood, S.A.; Pedrozo, F.; Varekamp, J.C.; Nelson, B.J.; Shope, C.L.; Baffico, G. Hydrogeochemistry and rare earth element behavior in a volcanically acidified watershed in Patagonia, Argentina. *Chem. Geol*. **2005**, *222*, 249–267.

34) Gammons, C.H.; Wood, S.A.; Nimick, D.A. Diel behavior of rare earth elements in a mountain stream with acidic to neutral pH. *Geochim. Cosmochim. Ac*. **2005**, *69*, 3747–3758.

35) Barroux, G.; Sonke, J.E.; Boaventura, G.; Viers, J.; Godderis, Y.; Bonnet, M.-P.; Sondag, F.; Gardoll, S.; Lagane, C.; Seyler, P. Seasonal dissolved rare earth element dynamics of the Amazon River main stem, its tributaries, and the Curuaí floodplain. *Geochem. Geophy. Geosy*. **2006**, *7*, Q12005.

36) Bau, M.; Knappe, A.; Dulski, P. Anthropogenic gadolinium as a micropollutant in river waters in Pennsylvania and in Lake Erie, northeastern United States. *Chem. Erde-Geochem.* **2006**, *66*, 143–152.

37) Kulaksiz, S.; Bau, M. Contrasting behaviour of anthropogenic gadolinium and natural rare earth elements in estuaries and the gadolinium input into the North Sea. *Earth Planet. Sc. Lett*. **2007**, *260*, 361–371.

38) Kulaksiz, S.; Bau, M. Anthropogenic gadolinium as a microcontaminant in tap water used as drinking water in urban areas and megacities. *Appl. Geochem.* **2011**, *26*, 1877–1885.

39) Pokrovsky, O.S.; Viers, J.; Shirokova, L.S.; Shevchenko, V.P.; Filipov, A.S.; Dupré, B. Dissolved, suspended, and colloidal fluxes of organic carbon, major and trace elements in the Severnaya Dvina River and its tributary. *Chem. Geol*. **2010**, *273*, 136–149.

40) Censi, P.; Sposito, F.; Inguaggiato, C.; Zuddas, P.; Inguaggiato, S.; Venturi, M. Zr, Hf and REE distribution in river water under different ionic strength conditions. *Sci. Total Environ.* **2018**, *645*, 837–853.

41) Kalender, L.; Aytimur, G. REE geochemistry of Euphrates River, Turkey. *J. Chem.* **2016**, *1*, 1–13.

42) Smith, C.; Liu, X.-M. Spatial and temporal distribution of rare earth elements in the Neuse River, North Carolina. *Chem. Geol*. **2018**, *488*, 34–43.

43) Merschel, G.; Bau, M.; Schmidt, K.; Münker, C.; Dantas, E.L. Hafnium and neodymium isotopes and REY distribution in the truly dissolved, nanoparticulate/colloidal and suspended loads of rivers in the Amazon Basin, Brazil. *Geochim. Cosmochim. Ac*. **2017**, *213*, 383–399.

44) Smedley, P.L. The geochemistry of rare earth elements in groundwater from the Carnmenellis area, southwest England. *Geochim. Cosmochim. Ac*. **1991**, *55*, 2767–2779.

45) Johannesson, K.H.; Lyons, W.B.; Stetzenbach, K.J.; Byrne, R.H. The solubility control of rare earth elements in natural terrestrial waters and the significance of PO_4^{3-} and CO_3^{2-} in limiting dissolved rare earth concentrations: a review of recent information. *Aquat. Geochem.* **1995**, *1*, 157–173.

46) Johannesson, K.H.; Stetzenbach, K.J.; Hodge, V.F. Rare earth elements as geochemical tracers of regional groundwater mixing. *Geochim. Cosmochim. Ac*. **1997**, *61*, 3605–3618.

47) Johannesson, K.H.; Farnham, I.M.; Guo, C.; Stetzenbach, K.J. Rare earth element fractionation and concentration variations along a groundwater flow path within a shallow, basin-fill aquifer, southern Nevada, USA. *Geochim. Cosmochim. Ac*. **1999**, *63*, 2697–2708.

48) Leybourne, M.I.; Goodfellow, W.D.; Boyle, D.R.; Hall, G.M. Rapid development of negative Ce anomalies in surface waters and contrasting REE patterns in groundwaters associated with Zn-Pb massive sulphide deposits. *Appl. Geochem.* **2000**, *15*, 695–723.

49) Tang, J.; Johannesson, K.H. Controls on the geochemistry of rare earth elements along a groundwater flow path in the Carrizo Sand aquifer, Texas, USA. *Chem. Geol*. **2006**, *225*, 156–171.

50) Esmaeili-Vardanjani, M.; Shamsipour-Dehkordi, R.; Eslami, A.; Moosaei, F.; Pazand, K. A study of differentiation pattern and rare earth elements migration in geochemical and hydrogeochemical environments of Airekan and Cheshmeh Shotori areas (Central Iran). *Environ. Earth Sci.* **2013**, *68*, 719-732.

51) Chevis, D.A.; Johannesson, K.H.; Burdige, D.J.; Tang, J.; Moran, S.B.; Kelly, R.P. Submarine groundwater discharge of rare earth elements to a tidally-mixed estuary in Southern Rhode Island. *Chem. Geol*. **2015**, *397*, 128–142.

52) Liu, H.; Guo, H.; Xing, L.; Zhan, Y.; Li, F.; Shao, J.; Niu, H.; Liang, X.; Li, C. Geochemical behaviors of rare earth elements in groundwater along a flow path in the North China Plain. *J. Asian Earth Sci.* **2016**, *117*, 33–51.

53) Johannesson, K.H.; Lyons, W.B. Rare-earth element geochemistry of Colour Lake, an acidic freshwater lake on Axel Heiberg Island, Northwest Territories, Canada. *Chem. Geol*. **1995**, *119*, 209–223.

54) De Carlo, E.H.; Green W.J. Rare earth elements in the water column of Lake Vanda, McMurdo Dry Valleys, Antarctica. *Geochim. Cosmochim. Ac*. **2002**, *66*, 1323–1333.

55) Sholkovitz, E.R. The geochemistry of rare earth elements in the Amazon River estuary. *Geochim. Cosmochim. Ac*. **1993**, *57*, 2181–2190.

56) Nozaki, Y.; Lerche, D.; Alibo, D.S.; Tsutsumi, M. Dissolved indium and rare earth elements in three Japanese rivers and Tokyo Bay: evidence for anthropogenic Gd and In. *Geochim. Cosmochim. Ac*. **2000**, *64*, 3975–3982.

57) Rousseau, T.C.C.; Sonke, J.E.; Chmeleff, J.; van Beek, P.; Souhat, M.; Boaventura, G.; Seyler, P.; Jeandel, C. Rapid neodymium release to marine waters from lithogenic sediments in the Amazon estuary. *Nat. Commun.* **2015**, *6*, 7592.

58) Elderfield, H.; Sholkovitz, E.R. Rare earth elements in the pore waters of reducing nearshore sediments. *Earth Planet. Sc. Lett*. **1987**, *82*, 280–288.

59) Bayon, G.; Birot, D.; Ruffine, L.; Caprais, J.-C.; Ponzevera, E.; Bollinger, C.; Donval, J.-P.; Charlou, J.-L.; Voisset, M.; Grimaud, S. Evidence for intense REE scavenging at cold seeps from the Niger Delta margin. *Earth Planet. Sc. Lett*. **2011**, *312*, 443–452.

60) Elderfield, H.; Greaves, M.J. The rare earth elements in seawater. *Nature* **1982**, *296*, 214–219.

61) de Baar, H.J.W.; Bacon, M.P.; Brewer, P.G. Rare-earth distributions with a positive Ce anomaly in the Western North Atlantic Ocean. *Nature* **1983**, *301*, 324–327.

62) German, C.R.; Holliday, B.P.; Elderfield, H. Redox cycling of rare earth elements in the suboxic zone of the Black Sea. *Geochim. Cosmochim. Ac*. **1991**, *55*, 3553–3558.

63) Piepgras, D.J.; Jacobsen, S.B. The behavior of rare earth elements in seawater: precise determination of variations in the North Pacific water column. *Geochim. Cosmochim. Ac*. **1992**, *56*, 1851–1862.

64) Sholkovitz, E.R.; Landing, W.M.; Lewis, B.L. Ocean particle chemistry: the fractionation of rare earth elements between suspended particles and seawater. *Geochim. Cosmochim. Ac*. **1994**, *58*, 1567–1579.

65) German, C.R.; Masuzawa, T.; Greaves, M.J.; Elderfield, H.; Edmond, J.M. Dissolved rare earth elements in the Southern Ocean: cerium oxidation and the influence of hydrography. *Geochim. Cosmochim. Ac*. **1995**, *59*, 1551–1558.

66) Zhang, J.; Nozaki, Y. Rare earth elements and yttrium in seawater: ICP-MS determinations in the East Caroline, Coral Sea, and South Fiji basins of the western South Pacific Ocean. *Geochim. Cosmochim. Ac*. **1996**, *60*, 4631–4644.

67) Hongo, Y.; Obata, H.; Alibo, D.S.; Nozaki, Y. Spatial variations of rare earth elements in North Pacific surface water. *J. Oceanogr.* **2006**, *62*, 441–455.

68) Wang, Z.-L.; Yamada, M. Geochemistry of dissolved rare earth elements in the Equatorial Pacific Ocean. *Environ. Geol.* **2007**, *52*, 779–787.

69) van de Flierdt, T.; Pahnke, K.; Amakawa, H.; Andersson, P.; Basak, C.; Coles, B.; Colin, C.; Crocket, K.; Frank, M.; Frank, N.; Goldstein, S.L.; Goswami, V.; Haley, B.A.; Hathorne, E.C.; Hemming, S.R.; Henderson, G.M.; Jeandel, C.; Jones, K.; Kreissig, K.; Lacan, F.; Lambelet, M.; Martin, E.E.; Newkirk, D.R.; Obata, H.; Pena, L.; Piotrowski, A.M.; Pradoux, C.; Scher, H.D.; Schöberg, H.; Singh, S.K.; Stichel, T.; Tazoe, H.; Vance, D.; Yang, J. GEOTRACES intercalibration of neodymium isotopes and rare earth element concentrations in seawater and suspended particles. Part 1: reproducibility of results for the international intercomparison. *Limnol. Oceanogr. Methods* **2012**, *10*, 234–251.

70) Grenier, M.; Jeandel, C.; Lacan, F.; Vance, D.; Venchiarutti, C.; Cros, A.; Cravatte, S. From the subtropics to the central equatorial Pacific Ocean: neodymium isotopic composition and rare earth element concentration variations. *J. Geophys. Res.-Oceans* **2013**, *118*, 592–618.

71) Jeandel, C.; Delattre, H.; Grenier, M.; Pradoux, C.; Lacan, F. Rare earth element concentrations and Nd isotopes in the Southeast Pacific Ocean. *Geochem. Geophy. Geosy.* **2013**, *14*, 328–341.

72) Garcia-Solsona, E.; Jeandel, C.; Labatut, M.; Lacan, F.; Vance, D.; Chavagnac, V.; Pradoux, C. Rare earth elements and Nd isotopes tracing water mass mixing and particle-seawater interactions in the SE Atlantic. *Geochim. Cosmochim. Ac*. **2014**, *125*, 351–372.

73) Abbott, A.N.; Haley, B.A.; McManus, J.; Reimers, C.E. The sedimentary flux of dissolved rare earth elements to the ocean. *Geochim. Cosmochim. Ac*. **2015**, *154*, 186–200.

74) Hathorne, E.C.; Stichel, T.; Brück, B.; Frank, M. Rare earth element distribution in the Atlantic sector of the Southern Ocean: the balance between particle scavenging and vertical supply. *Mar. Chem.* **2015**, *177*, 157–171.

75) Zheng, X.-Y.; Plancherel, Y.; Saito, M.A.; Scott, P.M.; Henderson, G.M. Rare earth elements (REEs) in the tropical South Atlantic and quantitative deconvolution of their non-conservative behavior. *Geochim. Cosmochim. Ac*. **2016**, *177*, 217–237.

76) Johannesson, K.H.; Palmore, C.D.; Fackrell, J.; Prouty, N.G.; Swarzenski, P.W.; Chevis, D.A.; Telfeyan, K.; White, C.D.; Burdige, D.J. Rare earth element behavior during groundwater-seawater mixing along the Kona Coast of Hawaii. *Geochim. Cosmochim. Ac*. **2017**, *198*, 229–258.

77) Osborne, A.H.; Hathorne, E.C.; Schijf, J.; Plancherel, Y.; Böning, P.; Frank, M. The potential of sedimentary foraminiferal rare earth element patterns to trace water masses in the past. *Geochem. Geophy. Geosy*. **2017**, *18*, 1550–1568.

78) de Baar, H.J.W.; Bruland, K.W.; Schijf, J.; van Heuven, S.M.A.C.; Behrens, M.K. Low cerium among the dissolved rare earth elements in the central North Pacific Ocean. *Geochim. Cosmochim. Ac*. **2018**, *236*, 5–40.

79) Haley, B.A.; Klinkhammer, G.P.; McManus, J. Rare earth elements in pore waters of marine sediments. *Geochim. Cosmochim. Ac*. **2004**, *68*, 1265–1279.

80) Kim, J.-H.; Torres, M.E.; Haley, B.A.; Kastner, M.; Pohlman, J.W.; Riedel, M.; Lee, Y.J. The effect of diagenesis and fluid migration on rare earth element distribution in fluids of the northern Cascadia accretionary margin. *Chem. Geol*. **2011**, *291*, 152–165.

81) Metzger, C.A.; Terry, D.O.; Grandstaff, D.E. Effect of paleosol formation on rare earth element signatures in fossil bone. *Geology* **2004**, *32*, 497–500.
